# Prescription of Ninjin’yoeito, A Japanese Kampo Medicine, in Relation to Levels of Long-Term Care Needs or Disabilities in Older Adults: A Cross-Sectional Study

**DOI:** 10.31662/jmaj.2025-0567

**Published:** 2026-04-03

**Authors:** Yui Sasaki, Reina Taguchi, Rumiko Tsuchiya-Ito, Satomi Kitamura, Masao Iwagami, Nobuo Sakata, Ichiro Arai, Yoshiharu Motoo, Shota Hamada

**Affiliations:** 1 Faculty of Pharmaceutical Sciences, Nihon Pharmaceutical University, Tokyo, Japan; 2 Research Department, Institute for Health Economics and Policy, Association for Health Economics Research and Social Insurance and Welfare, Tokyo, Japan; 3 Department of Education and Innovation Training for Pharmacy, National Center for Geriatrics and Gerontology, Aichi, Japan; 4 Research Department, Dia Foundation for Research on Ageing Societies, Tokyo, Japan; 5 UTokyo Nursing Dissemination and Implementation Science Institute, Tokyo, Japan; 6 Department of Gerontological Home Care and Long-term Care Nursing/Palliative Care Nursing, School of Health Sciences and Nursing, Graduate School of Medicine, The University of Tokyo, Tokyo, Japan; 7 Health Services Research and Development Center, University of Tsukuba, Ibaraki, Japan; 8 Heisei Medical Welfare Group Research Institute, Tokyo, Japan; 9 Department of Internal Medicine, Fukui Saiseikai Hospital, Fukui, Japan; 10 Department of Pharmacoepidemiology, School of Pharmacy, Tokyo University of Pharmacy and Life Sciences, Tokyo, Japan

**Keywords:** disability, frailty, herbal medicine, Kampo, long-term care, ninjin’yoeito

## Abstract

**Introduction:**

Ninjin’yoeito, a Japanese Kampo medicine, is expected to be effective for frailty, but its utilization in Japanese clinical practice is unknown. Thus, this study aimed to characterize older adults who were prescribed ninjin’yoeito in terms of long-term care (LTC) needs, physical and cognitive disabilities, and diagnoses.

**Methods:**

We conducted a population-based cross-sectional study using medical and LTC claims data for adults aged ≥75 years in 2019 in Hachioji City, Tokyo, Japan. LTC needs levels were classified into four categories: independent (including no certification), support level 1 or 2, care level 1 or 2, and care levels 3 to 5. Disability levels were categorized into nine groups based on the combination of levels of physical disability and cognitive impairment, which were assessed using nationally standardized measures at LTC needs certification. Multivariable logistic regression analyses were performed to evaluate the associations between ninjin’yoeito prescriptions during a three-month period and LTC needs or disability levels, adjusting for age and sex. We described the differences in the frequencies of International Classification of Diseases, Tenth Revision (ICD-10)-based diagnoses between participants with and without ninjin’yoeito prescriptions.

**Results:**

The study included 59,133 participants (median age, 80 years; 58.6% female). Ninjin’yoeito prescription prevalence was 0.22% (n = 130). Participants at support level 1 or 2, those at care level 1 or 2, and those who were independent living and had mild cognitive impairment were more likely to be prescribed ninjin’yoeito (adjusted odds ratio, 2.68; 95% confidence interval, 1.70-4.21; 1.91, 1.18-3.10; and 3.24, 2.04-5.15, respectively). Participants prescribed ninjin’yoeito were more likely to have diagnoses, including symptoms and signs related to food and fluid intake, malaise and fatigue, and dorsalgia.

**Conclusions:**

We identified the characteristics of community-dwelling older adults prescribed ninjin’yoeito. Our results may suggest that ninjin’yoeito was generally prescribed to older adults with frailty, within the indications of this formulation on the Japanese label.

## Introduction

The population of individuals aged ≥75 years in Japan is expected to account for 18.8% of the total population by 2030 ^(1)^. Frailty, defined as a state of decreased physiological reserve and increased vulnerability to stressors, is associated with a high risk of adverse outcomes, including disability, hospitalization, and mortality ^(2)^. Among community-dwelling older adults, the prevalence of frailty has been reported to be 7.4%, with a marked increase as age advances, particularly among adults aged 75 years and older ^(3)^. Therefore, frailty is a growing public health concern ^(4)^, and projections indicate that it will substantially contribute to increased healthcare and long-term care (LTC) costs in Japan over the next two decades ^(5)^. The Asia-Pacific Clinical Practice Guidelines for the Management of Frailty recommend engaging in physical activity, particularly resistance training; reducing inappropriate or excessive medications; fatigue screening; and considering protein supplementation ^(6)^. Nonetheless, effective frailty interventions are limited.

Ninjin’yoeito, also known as Ren Shen Yang Rong Tang in Chinese and Insamyangyoung-tang in Korean, is a Kampo formula consisting of 12 crude drugs, covered under national health insurance, and available as an over-the-counter drug in Japan. Although it is frequently prescribed for fatigue and malaise ^(7)^, recently, ninjin’yoeito has attracted attention for its potential to manage age-related conditions. In non-clinical studies, ninjin’yoeito was demonstrated to improve age-related factors, such as bone density, muscle mass, physical performance, and self-care motivation ^(8), (9), (10)^. In clinical studies, despite relatively small sample sizes, ninjin’yoeito may exert beneficial effects against muscle weakness, frailty, neuropsychiatric symptoms, and cognitive decline in older adults ^(11), (12), (13), (14)^. Moreover, a clinical study showed that ninjin’yoeito was beneficial in preventing frailty ^(7)^, in addition to improving fatigue/malaise and anorexia. Therefore, ninjin’yoeito may help prevent frailty development and progression.

We previously reported that ninjin’yoeito prescriptions for older adults, especially those aged ≥80 years, increased between 2015 and 2020 ^(15)^, potentially suggesting the use of ninjin’yoeito for frailty, cognitive decline, or dementia. Nonetheless, the study used aggregated prescription data stratified only by age and sex, thereby limiting the understanding of the characteristics of older adults prescribed ninjin’yoeito. Therefore, this study aimed to address this gap by examining the association between ninjin’yoeito prescriptions and LTC needs or disability levels using individual-level medical and LTC claims data, thereby providing a basis for considering the appropriate use of ninjin’yoeito in older adults.

## Materials and methods

### Study design and setting

This cross-sectional study used medical and LTC claims data for residents aged ≥75 years in 2019 in Hachioji City, Tokyo, Japan. As of September 2019, the city had a population of approximately 560,000, of whom 13.5% were aged ≥75 years ^(16)^, a proportion comparable with the national average of 14.7% (as of October 2019) ^(17)^.

### Data sources and selection of study participants

We used medical claims, LTC claims, and LTC needs certification data obtained from the city. First, we selected participants aged ≥75 years who were covered by the health insurance system for older adults. Given that nearly all individuals in Japan are mandatorily covered by this public health insurance system upon reaching 75 years of age, regardless of health status (except for those receiving public assistance), this approach allowed us to include almost all residents aged ≥75 years in the city. Next, we selected those with unchanged LTC needs levels during a relatively short three-month period (i.e., September and November 2019) to investigate the prescribing of ninjin’yoeito among individuals with stable LTC needs levels.

Medical claims data included International Classification of Diseases, Tenth Revision (ICD-10) diagnoses, prescriptions, and hospital and outpatient services. The LTC claims data included both home and facility-based services. The LTC needs certification data included LTC needs levels and nationally standardized assessments of physical disability and cognitive impairment. All data were anonymized by assigning unique identifiers to each insured individual to enable linkage between medical and LTC claims.

### Measures

Age, sex, and levels of LTC needs, physical disability, or cognitive impairment were determined in September 2019. LTC needs certification is mandatory for the use of LTC services under the public LTC insurance system and is based on standardized nationwide assessment tools ^(18)^. All ICD-10 diagnoses, except for suspected diagnoses, were examined during the three-month observation period from September to November 2019. Ninjin’yoeito prescriptions were identified from outpatient and pharmacy claims, regardless of dosage or duration.

The latest data on the levels of LTC needs, physical disability, or cognitive impairment that had been assessed at the time of LTC needs certification were used. LTC needs levels were categorized into four groups: independent (including no certification), support level 1 or 2, care level 1 or 2, and care levels 3 to 5 (most dependent). Physical disability levels, assessed using the “Independence in Daily Living for Older People with Disabilities,” were categorized into three groups: (1) independent; (2) independent living (rank J: some disability but largely independent and able to go out unassisted); or (3) semi-bedridden/bedridden (rank A, B, or C: requiring partial to complete assistance with daily life) ^(19)^. Cognitive impairment levels, assessed using the “Independence in Daily Living for Older People with Dementia,” were categorized into three groups: (1) independent; (2) mild/moderate (rank I or II: independent for daily life if watched by someone but having daily life-disturbing symptoms, behaviors, and problems in communication); or (3) severe (rank III, IV, or M: requiring assistance or expert management due to marked psychiatric symptoms) ^(20)^. Furthermore, participants were categorized into nine groups based on the combinations of levels of physical disability and cognitive impairment.

### Statistical analysis

Descriptive statistics were used to summarize the participants’ characteristics and the prevalence of ninjin’yoeito prescriptions. To explore potential ninjin’yoeito indications, the frequencies of recorded diagnoses were compared between individuals prescribed ninjin’yoeito and individuals not prescribed ninjin’yoeito. Multivariable logistic regression analysis was used to evaluate the association between ninjin’yoeito prescriptions (coded as prescribed = 1 and not prescribed = 0) and the level of LTC needs or the combinations of levels of physical disability and cognitive impairment. The results are reported as adjusted odds ratios (aORs) with 95% confidence intervals (CIs), adjusted for age (75-79, 80-89, and ≥90 years) and sex (male or female). Interaction terms were not included in the models. The goodness of fit of the logistic regression models was assessed using the Hosmer-Lemeshow test. All analyses were performed using Stata version 17 (StataCorp, College Station, TX, USA).

## Results

### Study participant characteristics

The study included 59,133 participants with a median age of 80 years (interquartile range: 77-85), and 58.6% were female (Table 1). Regarding LTC needs levels, 10.7%, 12.9%, and 6.5% were at support level 1 or 2, care level 1 or 2, and care levels 3 to 5, respectively. Regarding physical disability, 10.5% and 19.6% were categorized as independent living and semi-bedridden/bedridden, respectively. Regarding cognitive impairment, 22.6% and 4.2% were categorized as mild/moderate and severe, respectively.

**Table 1.** Study participant characteristics (N = 59,133).

		n (%) / median [IQR]
Age (years)		80 [77-85]
Age group (years)	75-79	26,244 (44.4)
	80-89	27,722 (46.9)
	≥90	5,167 (8.7)
Sex	Female	34,652 (58.6)
LTC needs levels	Independent	41,310 (69.9)
	Support level 1 or 2	6,331 (10.7)
	Care level 1 or 2	7,653 (12.9)
	Care levels 3 to 5	3,839 (6.5)
Physical disability^a^	Independent	41,317 (69.9)
	Independent living (rank J)	6,223 (10.5)
	Semi-bedridden/bedridden (rank A, B, or C)	11,593 (19.6)
Cognitive impairment^b^	Independent	43,328 (73.3)
	Mild/moderate (rank I or II)	13,335 (22.6)
	Severe (rank III, IV, or M)	2,470 (4.2)

IQR: interquartile range; LTC: long-term care. a. Based on the “Independence in Daily Living for Older People with Disabilities” b. Based on the “Independence in Daily Living for Older People with Dementia”

### Prescription of ninjin’yoeito

During the three-month observation period, 130 participants (0.22% of 59,133) received ninjin’yoeito prescriptions. The prevalence was highest among those who were independent living and had mild/moderate cognitive impairment (0.57%), followed by those at support level 1 or 2 (0.47%) (Table 2).

**Table 2.** Association of Levels of Long-Term Care Needs or Disability with Ninjin’yoeito Prescription.

	n	N	%	aOR (95% CI)	p-Value
**LTC needs levels**				
Independent	65	41,310	0.16	Reference	Reference
Support level 1 or 2	30	6,331	0.47	2.68 (1.70-4.21)	<0.001
Care level 1 or 2	26	7,653	0.34	1.91 (1.18-3.10)	0.009
Care levels 3 to 5	<10	3,839		1.29 (0.62-2.68)	0.497
*Hosmer-Lemeshow χ^2^ (df = 5)*				*2.13*	*0.831*
**Disability levels (physical disability^a^ and cognitive impairment^b^)**				
Independent and independent	65	41,313	0.16	Reference	Reference
Independent and mild/moderate	<10	<10		-	-
Independent and severe	<10	<10		-	-
Independent living and independent	<10	1,270		1.82 (0.66-5.03)	0.248
Independent living and mild/moderate	28	4,895	0.57	3.24 (2.04-5.15)	<0.001
Independent living and severe	<10	58		-	-
Semi-bedridden/bedridden and independent	<10	745		1.57 (0.38-6.46)	0.530
Semi-bedridden/bedridden and mild/moderate	24	8,437	0.28	1.58 (0.96-2.61)	0.075
Semi-bedridden/bedridden and severe	<10	2,411		1.57 (0.70-3.54)	0.277
*Hosmer-Lemeshow χ^2^ (df = 6)*				*2.17*	*0.903*

aOR: adjusted odds ratio; CI: confidence interval; LTC: long-term care. Masked where there were fewer than 10 participants. ^a^Based on the “Independence in Daily Living for Older People with Disabilities” ^b^Based on the “Independence in Daily Living for Older People with Dementia”

Ninjin’yoeito prescription was associated with support level 1 or 2 (aOR, 2.68; 95% CI, 1.70-4.21) and care level 1 or 2 (aOR, 1.91; 95% CI, 1.18-3.10) (Table 2). Ninjin’yoeito prescription was also associated with independent living and mild/moderate cognitive impairment (aOR, 3.24; 95% CI, 2.04-5.15). The Hosmer-Lemeshow test showed no evidence of lack of fit (χ^2^ = 2.13, df = 5, p = 0.831 for the model with LTC needs levels and χ^2^ = 2.17, df = 6, p = 0.903 for the model with disability levels).

### Recorded diagnoses

The top 10 ICD-10 codes ranked by the largest differences in diagnosis rates between those prescribed ninjin’yoeito and those not prescribed ninjin’yoeito are shown in Table 3. The diagnosis showing the largest differences included symptoms and signs related to food and fluid intake, such as anorexia and feeding difficulties (ICD-10 R63) (prescribed, 43.8% vs not prescribed, 3.2%); malaise and fatigue (ICD-10 R53) (prescribed, 25.4% vs not prescribed, 1.1%); and dorsalgia, including cervicalgia and lumbago with sciatica (ICD-10 M54) (prescribed, 45.4% vs not prescribed, 28.8%). Among those prescribed ninjin’yoeito, the most frequent diagnoses were other functional intestinal disorders, such as functional diarrhea and constipation (ICD-10 K59, 53.1%); gastritis and duodenitis (ICD-10 K29, 51.5%); and dorsalgia (ICD-10 M54, 45.4%).

**Table 3.** Top 10 ICD-10 Diagnoses with the Large Differences in Diagnosis Rates According to the Presence or Absence of Ninjin’yoeito Prescriptions.

ICD-10	Diseases	Prescribed (N = 130)	Not prescribed (N = 59,003)
		n (%)	n (%)
R63	Symptoms and signs concerning food and fluid intake	57 (43.8)	1,889 (3.2)
R53	Malaise and fatigue	33 (25.4)	669 (1.1)
M54	Dorsalgia	59 (45.4)	17,010 (28.8)
K59	Other functional intestinal disorders	69 (53.1)	22,418 (38.0)
M81	Osteoporosis without pathological fracture	56 (43.1)	16,642 (28.2)
M47	Spondylosis	45 (34.6)	12,419 (21.0)
K29	Gastritis and duodenitis	67 (51.5)	22,650 (38.4)
I50	Heart failure	44 (33.8)	12,377 (21.0)
J30	Vasomotor and allergic rhinitis	47 (36.2)	13,826 (23.4)
G47	Sleep disorders	56 (43.1)	18,134 (30.7)

ICD-10: International Classification of Diseases, Tenth Revision.

## Discussion

The study aimed to clarify the utilization of ninjin’yoeito in Japanese clinical practice by describing the characteristics of older adults who were prescribed ninjin’yoeito. Given that prescriptions of ninjin’yoeito are not frequent in the general population ^(15), (21)^, the use of the large-scale claims database in this study enabled us to characterize the profiles of older adults who received ninjin’yoeito. We found that participants at support level 1 to care level 2, as well as those who were independent living and had mild/moderate cognitive impairment, were more likely to be prescribed ninjin’yoeito. Such individuals often exhibit functional decline without severe disability, possibly corresponding to frailty ^(22)^. We speculate that physicians prescribe ninjin’yoeito to prevent further deterioration of physical function.

Possible medical backgrounds for ninjin’yoeito prescriptions included symptoms related to food and fluid intake and malaise/fatigue, consistent with the Japanese labeling information for ninjin’yoeito. Ninjin’yoeito is traditionally indicated for symptoms such as fatigue, anorexia, and general weakness, which are frequently accompanied by frailty ^(11)^. Its pharmacological actions, such as improving appetite and promoting cerebral blood flow, may contribute to better nutritional status and physical resilience ^(23), (24)^. Furthermore, the link between physical frailty and cognitive decline, as demonstrated in a longitudinal study ^(22)^, may suggest that ninjin’yoeito can indirectly benefit cognitive health by mitigating frailty-related pathways.

Our results are also supported, at least partly, by the findings from previous clinical studies that showed the potential beneficial effects of ninjin’yoeito on frailty and related conditions. Across different older adult populations, including those with chronic obstructive pulmonary disease (COPD) and Alzheimer’s disease, ninjin’yoeito has consistently been suggested to improve fatigue and anorexia ^(7), (13), (14), (25)^. In addition, improvements in knee extension strength have been reported in patients with COPD and fatigue ^(13)^. Although these findings are primarily based on before-after comparisons, improvements have also been observed in several domains of the Kihon Checklist, a brief screening tool for frailty, including activities of daily living, motor function, oral function, and depression ^(7)^; in walking speed and skeletal muscle mass ^(13)^, and in Mini-Mental State Examination scores in patients with Alzheimer’s disease ^(14)^. Taken together, these findings suggest that ninjin’yoeito may contribute to the improvement or reversal of frailty through enhancements in muscle strength, nutritional status, and cognitive function. The higher prevalence of ninjin’yoeito prescribing among older adults with mild to moderate physical disability or cognitive impairment observed in our study may reflect physicians’ perceptions that these patients have potentially reversible health states (i.e., frail) and do not require more intensive interventions, such as pharmacological treatment or nutritional management. In contrast, based on its mechanisms of action, ninjin’yoeito might also be expected to be beneficial for patients with higher levels of care dependency or disability; however, it remains unclear whether these effects differ according to the degree of physical or cognitive disability and the reasons why ninjin’yoeito was not prescribed more frequently among more functionally dependent individuals in the present study. Therefore, ninjin’yoeito may represent a supportive therapy for conditions associated with frailty; however, further research is needed to understand how existing evidence has influenced prescribing patterns in clinical practice.

This study had some limitations. First, the physician’s intent to prescribe ninjin’yoeito was only estimated; it could not be determined using claims data. Second, LTC needs certification is not a comprehensive survey, and some individuals who did not apply for LTC needs certification may have been misclassified as individuals with physical or cognitive independence. Related to this issue, we focused on individuals whose LTC needs levels remained stable over a three-month period. Given that reassessment of LTC needs levels usually occurs at intervals of one year or longer and often does not result in changes in LTC needs levels, this restriction is unlikely to have substantially affected our results. Third, although this study included a large number of participants, odds ratios could not be calculated for some disability levels due to the low prevalence of ninjin’yoeito prescriptions. Moreover, the very small number of ninjin’yoeito prescriptions in certain categories may have compromised the stability of the regression estimates and introduced potential bias. These limitations should be taken into account when interpreting the findings. Fourth, over-the-counter ninjin’yoeito could not be captured using claims data. However, because the copayment rate for adults aged ≥75 years is generally 10%, making ninjin’yoeito relatively inexpensive when prescribed, the incentive to purchase over-the-counter ninjin’yoeito was likely low. Finally, although we used claims data from a relatively large city, the number of older adults prescribed ninjin’yoeito was limited. Consequently, the findings may have been influenced by the prescribing preferences of specific physicians or health care institutions, which may limit the generalizability of our findings to broader prescribing practices. In addition, because the data were collected in a single city in a country with public health insurance coverage for Kampo medicines, the findings may not be generalizable to other regions and countries.

In conclusion, we identified the characteristics of community-dwelling older adults prescribed ninjin’yoeito. Our results may suggest that ninjin’yoeito was generally prescribed to older adults with frailty, within the indications of this formulation on the Japanese label. As further evidence accumulates regarding the effectiveness and safety of ninjin’yoeito, its appropriate use should be explored in future studies.

## Article Information

### Acknowledgments

We sincerely thank the officials at Hachioji City Hall for providing the data for this study.

### Author Contributions

Yui Sasaki and Shota Hamada conceptualized and designed the study. Reina Taguchi, Rumiko Tsuchiya-Ito, Satomi Kitamura, and Shota Hamada obtained data. Yui Sasaki and Shota Hamada performed the analyses. All authors interpreted the data. Yui Sasaki drafted the manuscript. Reina Taguchi, Rumiko Tsuchiya-Ito, Satomi Kitamura, Masao Iwagami, Nobuo Sakata, Ichiro Arai, Yoshiharu Motoo, and Shota Hamada critically reviewed and revised the manuscript. All authors have approved the final manuscript for submission.

### Conflicts of Interest

Shota Hamada belonged to an endowed chair funded by donations from Hakue Technology, PROUMED, Japan Bio Products, Towa Pharmaceutical, Yellow Eight, and Sugi Holdings. Ichiro Arai received payments from Tsumura & Co. outside of this work. Yoshiharu Motoo received lecture fees from Tsumura & Co. The other authors declare no conflicts of interest.

### Ethical Consideration

This study protocol was approved by the ethical review board of the Institute for Health Economics and Policy (R2-001).

### Data Availability Statement

The data that support the findings of this study are available from the corresponding author upon reasonable request.

### Informed Consent

The requirement for informed consent was waived due to the anonymous nature of the database.

### Consent for Publication

Not applicable.
